# Supplementary material for: Insights into the formation mechanism of two-dimensional lead halide nanostructures
Source: Nanoscale. 2018 Feb 9;10(9):4442–51. doi: 10.1039/c7nr09564c (PMC5831117; doi:10.1039/c7nr09564c)
Supplement: Supplementary file 1 [file NR-010-C7NR09564C-s001.pdf]

Electronic Supplementary Material (ESI) for Nanoscale.
This journal is © The Royal Society of Chemistry 2018

Insights into the formation mechanism of two-dimensional lead halide nanostructures

Supporting information

Eugen Klein[1], Rostyslav Lesyuk[1,2], Christian Klinke[1,3,*]

[1] Institute of Physical Chemistry, University of Hamburg,
Grindelallee 117, 20146 Hamburg, Germany
[2] Pidstryhach Institute for applied problems of mechanics and mathematics of NAS of Ukraine,
Naukowa str. 3b, 79060 Lviv, Ukraine
[3] Department of Chemistry, Swansea University - Singleton Park, Swansea SA2 8PP, UK

* Corresponding author: klinke@chemie.uni-hamburg.de

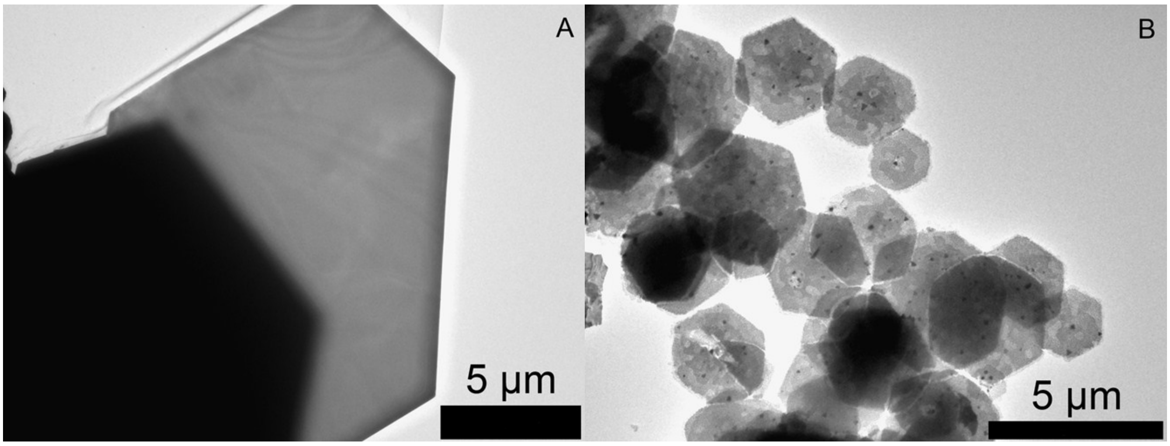

Figure S1. TEM images of PbI_2 nanosheets. (A) Example for particles prepared at 200 °C without TOP. (B) Overview for particles obtained immediately after the formation of the sheets with a lateral size of 1 - 4 µm.

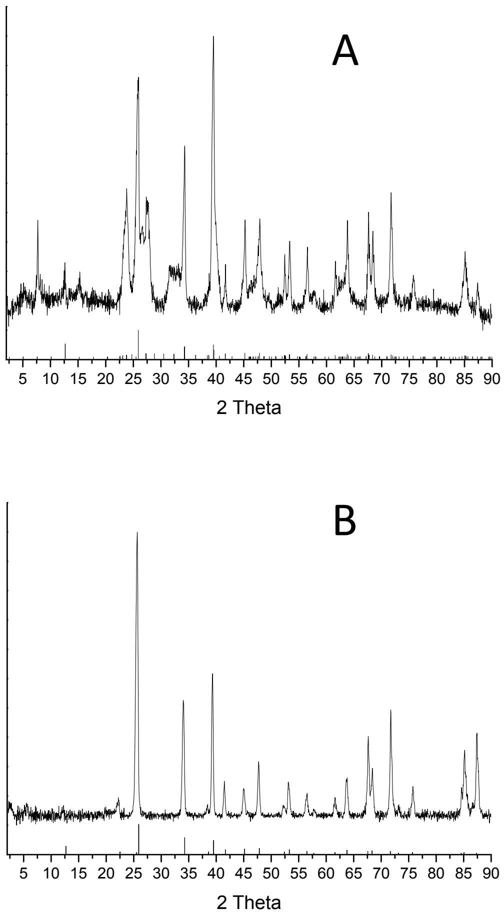

Figure S2. Powder XRD in a capillary for the growth time of (A) 0 min and (B) 1 h. The sheets synthesized after 1 h showing all the expected reflexes for the P-3m1 space group while those prepared right after the formation missing a high number of reflexes for the P3m1 space group respectively.

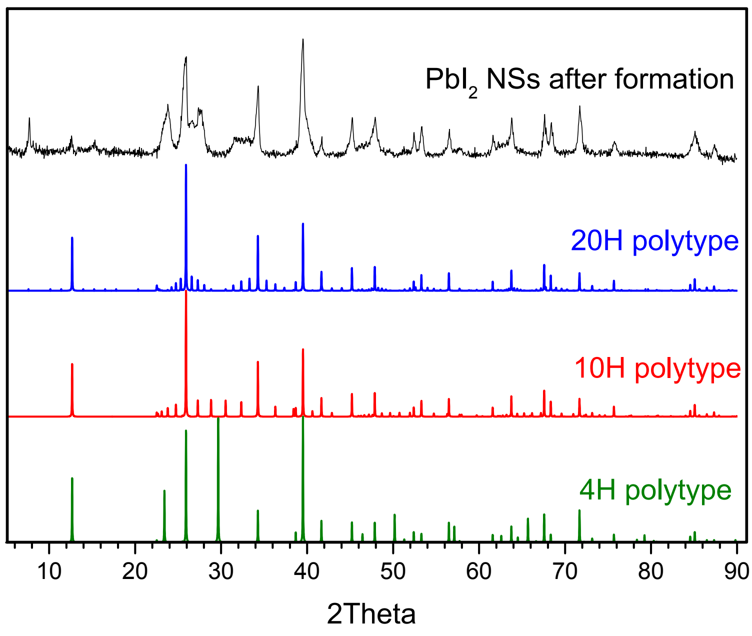

Figure S3. XRD of PbI_2 nanosheets recorded by the capillary method compared to simulated 20H, 10H and 4H powder XRD spectra (simulations performed according to the crystallographic data from Mitchel [33] and Agrawal et al. [37] in PowderCell software, the value z=0.265 was adapted from Mitchel for ion coordinates in c-direction).

The XRD matches well with the major reflexes of the 10H/20H structure: 25.9 °, 34.3 °, 39.5 °, 41.7 °, 45.3 °, 47.9 °, 52.4 °, 53.4 °, 56.5 °, 61.7 °, 63.8 °, 67.6 °, 68.5 °, 71.7 °, 75.7 °, 85.1 °. The intensity distribution of major reflexes fits the simulated spectra in general well, except of the 71.6 ° signal. It is possible to distinguish between 20H and 10H polytypes searching for minor reflexes. For example, the XRD signal intensity distribution around 27° – 27.8° obviously comes from reflexes at 26.6 ° (1 0 11), 27.3 ° (1 0 12) and 28 ° (1 0 13) of the 20H structure and the 27.3 ° reflex (016)/(106) of the 10H structure. In this case, the intensity of the 27.3 ° reflex will be more intense than 26.6 °, which is not the case of the pure 20H structure. XRD patterns of merely the 10H structure cannot explain the appearance of the 26.6 ° reflex, thus we consider the measured spectrum as superposition of these polytypes. The diffraction intensity distribution between 31 ° and 35 ° is another evidence of the overlay of diffraction signal from different polytypes. All of the considered polytypes produce the reflex at 34.4 ° (that is why it appears so intense), but minor reflexes are uniformly distributed in the angle region of 31–33.5 °. With transition to the 2H structure (P-3m1 space group) this intensity distribution disappears.

Questionable is the origin of the 23.8 ° reflex. Among hexagonal polytypes the only polytype which can explain the appearance of this reflex is the 4H polytype. There are several minor reflexes which could also be attributed to the 4H structure, like 46.4 ° for (021)/(201), 57.2 ° for (025)/(205) and 62.3 ° for (121)/(211). The intensification of the 71.6 ° reflex of the 20H may also come from the 4H signal (otherwise the 71.7 ° reflex would be less intense as 67.6°). However single reflexes at 29.7 ° (013)/(103) and 50.2 ° (023)/(203) of the 4H structure were not found in the XRD spectrum. The appearance of many polytypes in our crystals seems to be high starting from the NSs formation moment and to decrease with reaction time. Since the stability of NSs after 0–5 min synthesis is weak under ambient conditions (this is accompanied by stoichiometry changes), they quickly undergo the polytypic transitions and eventually become the 2H structure. That means, during the capillary measurement they can undergo structural changes and produce an XRD which is rather complex but can be explained if we consider the mixture of several polytypes PbI_2 NSs, including the 2H.

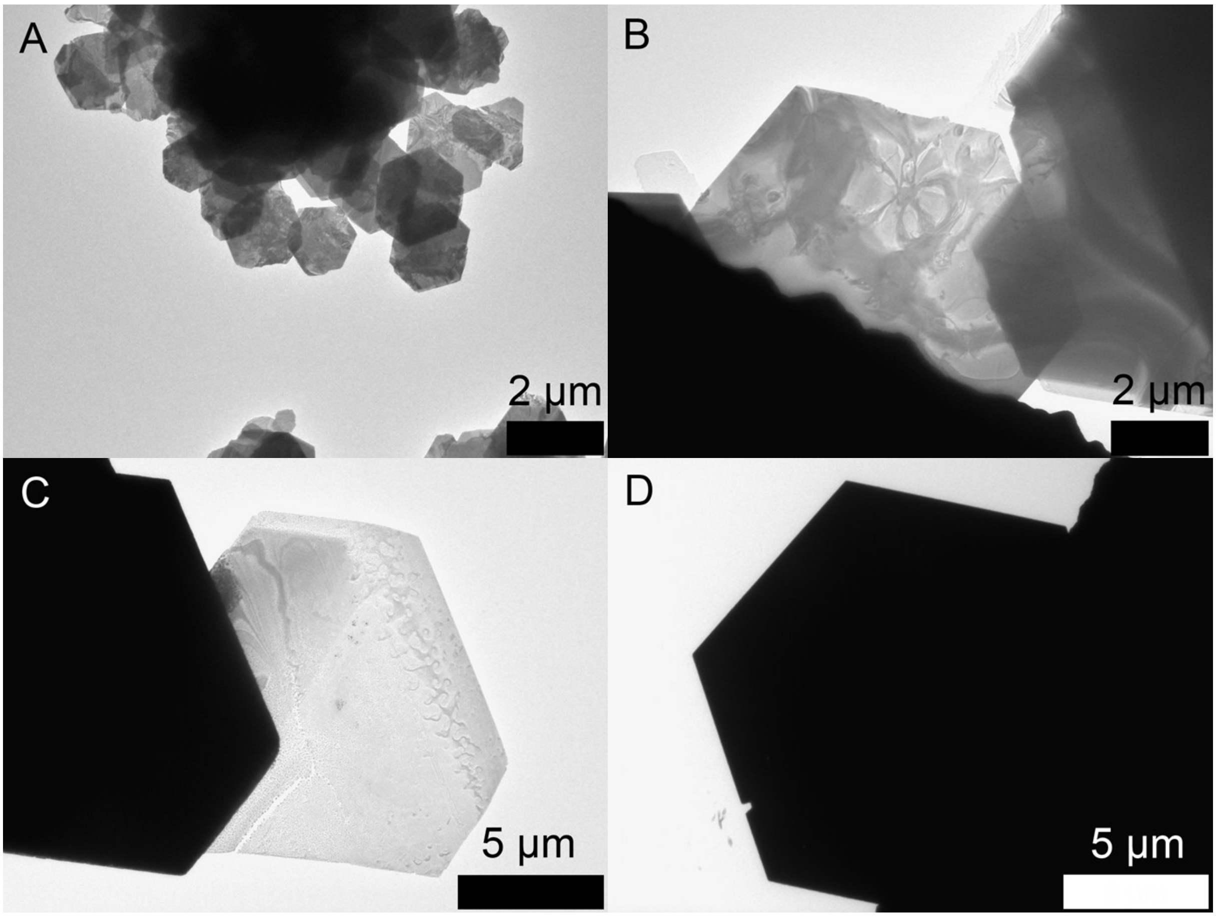

Figure S4. TEM images of PbI_2 nanosheets prepared by varying the temperature at synthesis parameters where the solvent has a ratio of 3:1 diphenyl ether to oleic acid. The temperature increases from 120 °C (A) to 150 °C (B) to 200 °C (C), and to 300°C (D) respectively. The lateral size is changing with the temperature from 2 µm to 7 µm to 10 µm and to finally 15 µm.

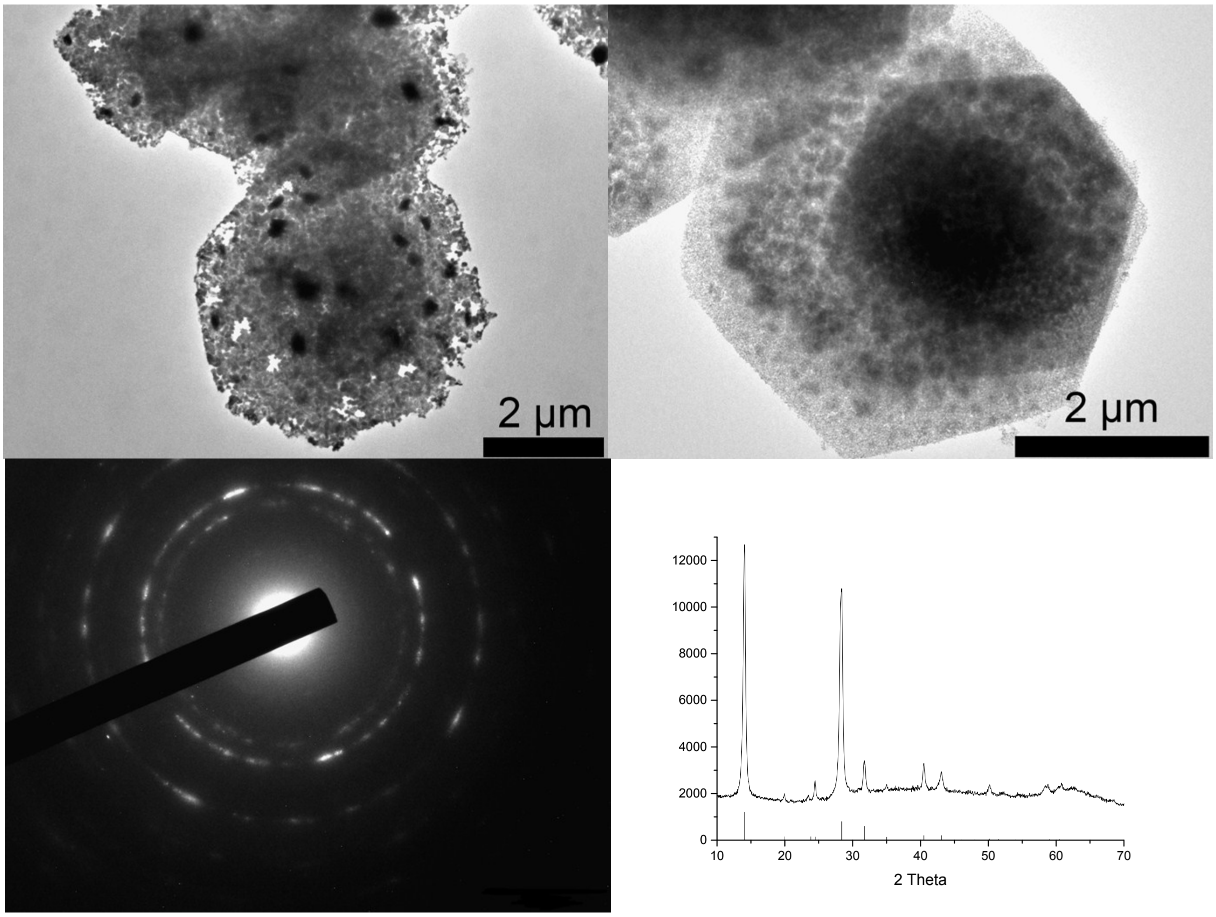

Figure S5. TEM images, SAED and XRD patterns of PbI_2 methylammonium iodide perovskites after the incorporation of the methylammonium iodide molecules in PbI_2 nanosheets.

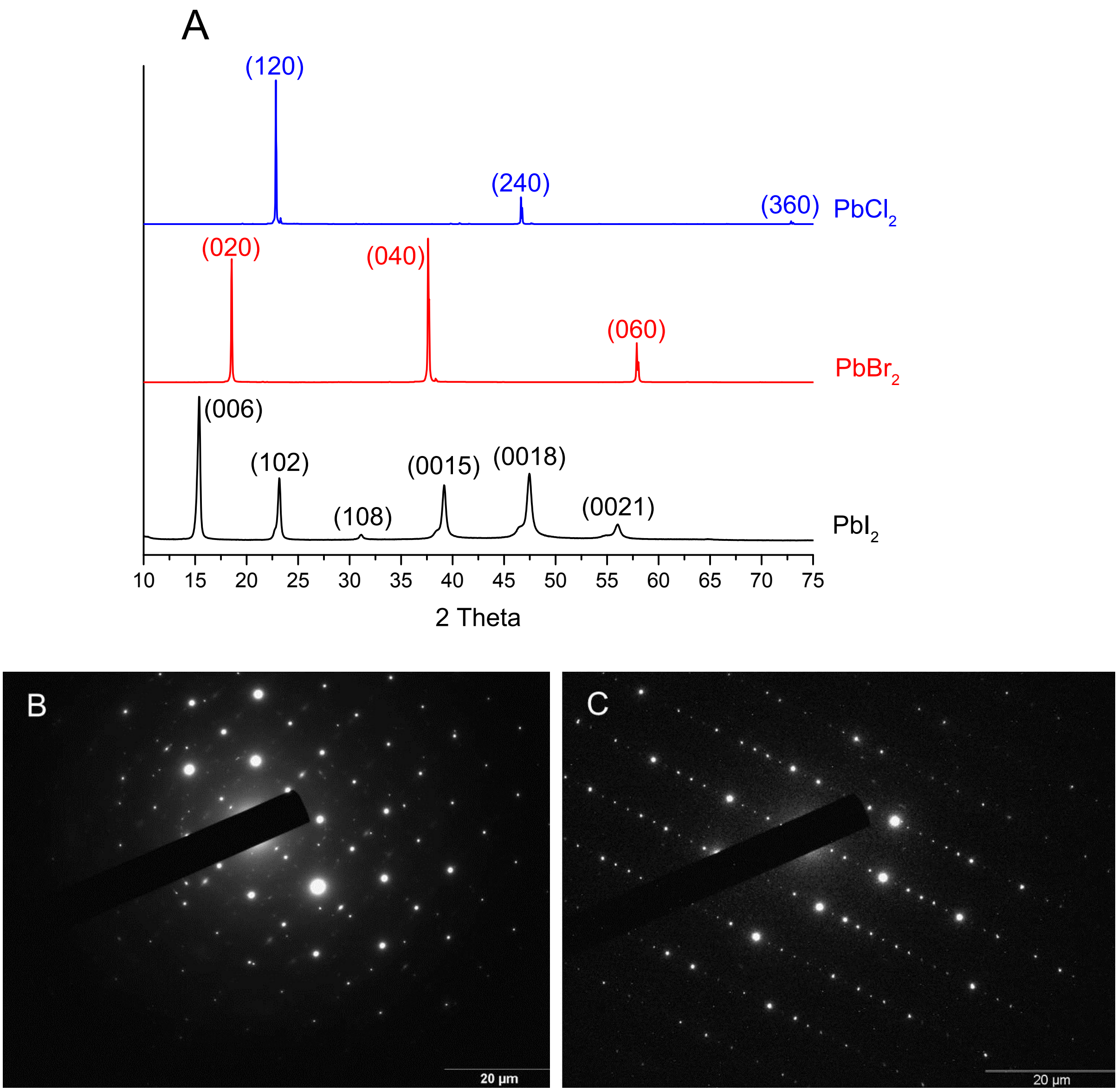

Figure S6. (A) Powder XRD of PbI_2, $PbBr_2$ and $PbCl_2$ nanosheets. All three examples showing the suppression of most of the signals, which indicates the preferred orientation of the nanosheets on the surface of the wafer along the lateral dimensions. (B) Electron diffraction (SAED) patterns of the $PbBr_2$ nanosheet. (C) SAED patterns of the $PbCl_2$ nanosheet.

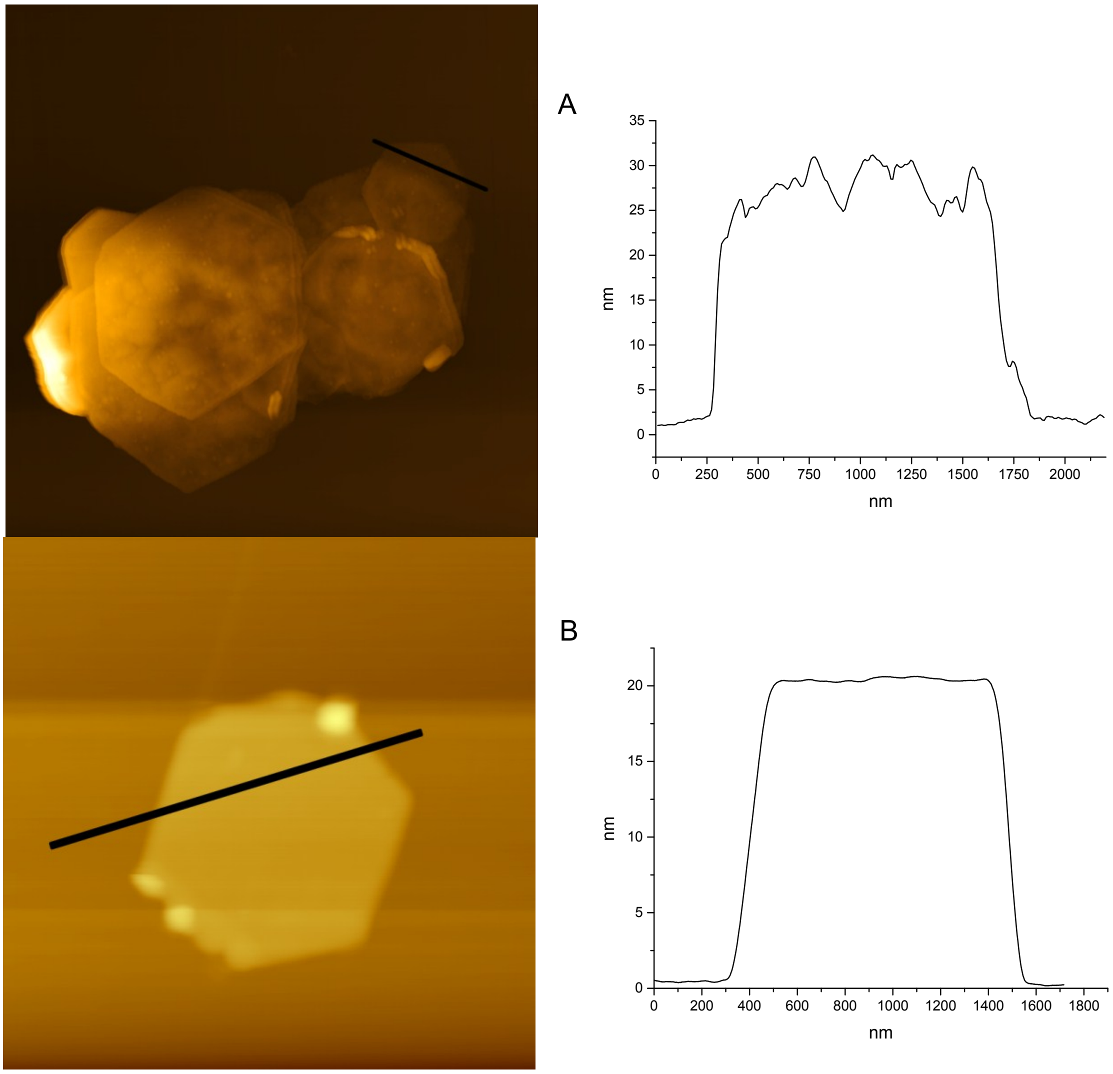

A
35
30
25
20
15
10
5
0
nm
0
250
500
750
1000
1250
1500
1750
2000
nm
B
20
15
10
5
0
nm
0
200
400
600
800
1000
1200
1400
1600
1800
nm

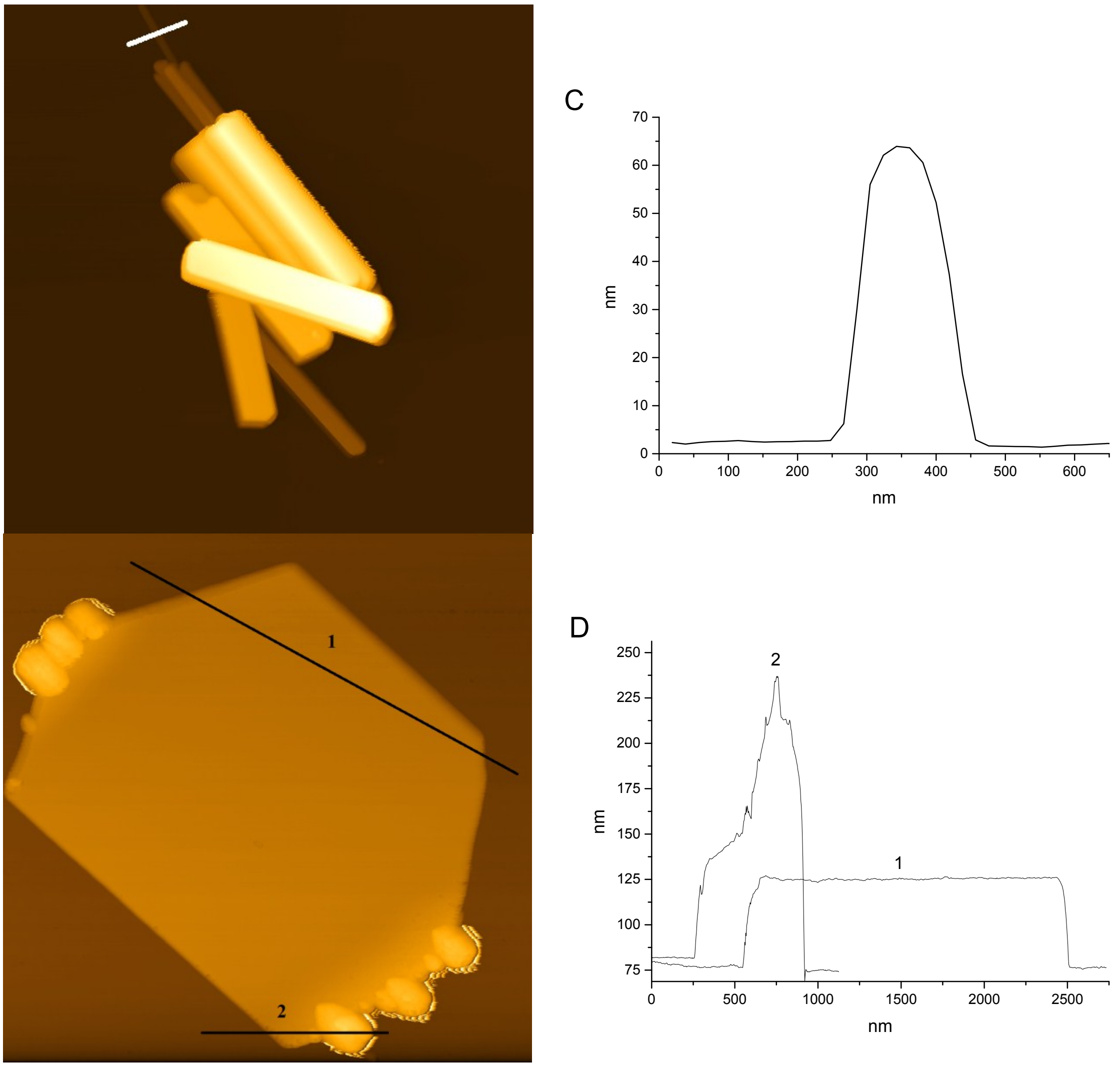

Figure S7. AFM images and height profiles of PbI_2, $PbBr_2$ and $PbCl_2$ nanosheets. They possess total heights of (A) 25 nm, (B) 20 nm, and (C) 63 nm. (D) $PbBr_2$ nanosheet from the same sample as the platelet shown in (B) with a thickness of 50 nm and on some edges 150 nm. The total heights include a top and a bottom layer of self-assembled OA of a thickness of about 1.8 nm.

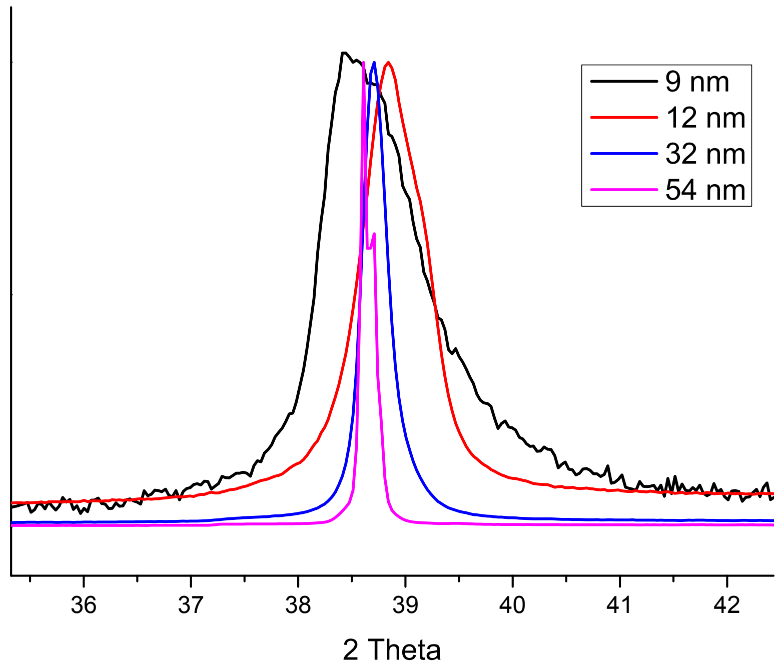

Figure S8. The peak at about 38 ° taken from the XRD data for PbI_2 nanosheets with different thicknesses. The thickness increases with narrower signal. 9 nm thick sheets prepared at 60 °C. 12 nm thick sheets synthesized at 80 °C and a 5 times higher DIE amount. 32 nm thick sheets prepared at 100 °C and 54 nm thick nanosheets prepared at 200 °C.

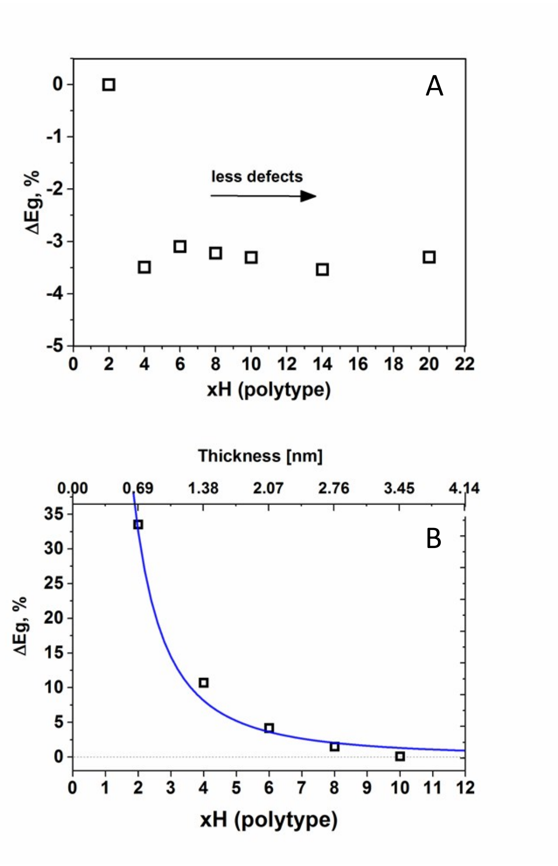

Figure S9. (A) Relative change (ΔE_g) of the band gap as a function of polytype in PbI_2. For structures different from 2H the ΔE_g is negative. (B) Relative change of the band gap in PbI_2 as a function of domain structure in confinement, which corresponds to the 2H, 4H, 8H and 10H polytypes (squares). Blue line denotes the ~$1/x^2$ fit. The top x-axis indicates the corresponding thickness of the structure in confinement.

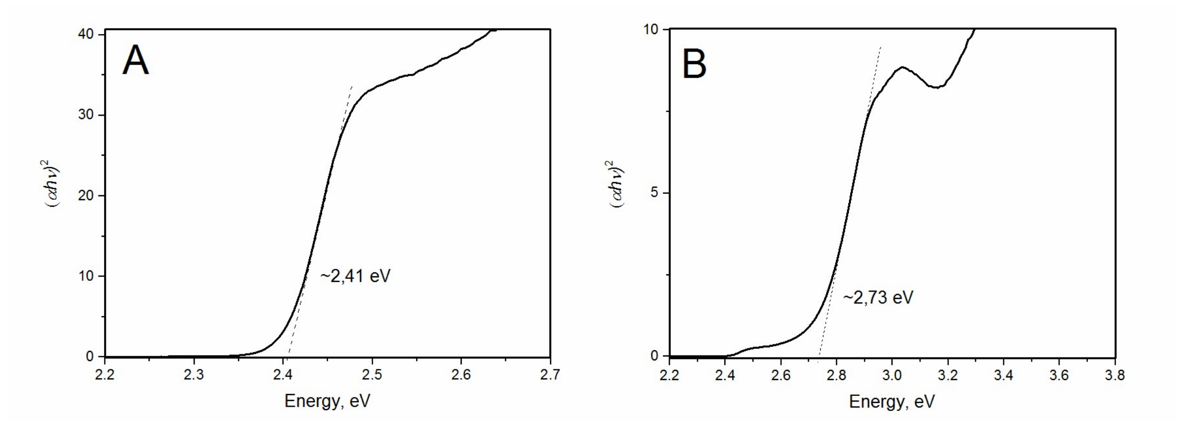

Figure S10. Intensity $(\alpha h\nu)^2$ as function of photon energy (Tauc plots) of PbI_2 nanosheets in chloroform: (A) after 1 hour synthesis (P$\bar{3}m1$ *space group*). The information about the effective band gap has been extracted. E_g(a)=2.73 eV, E_g(b)=2.41 eV. The shift corresponds to ~330 meV (13.8% increase for P3m1 space group sample); (B) after first minutes of the synthesis (P3m1 space group).

DFT calculations

Band structure simulations have been performed under periodic boundary conditions in the frame of the density functional theory (DFT) employing the ABINIT software package [S1,S2], a common project of the Université Catholique de Louvain, Corning Incorporated, and other contributors (URL http://www.abinit.org). The calculations were performed with the Perdew-Burke-Ernzerhof GGA functional [S3,S4] and Hartwigsen-Goedecker-Hutter pseudopotentials [S5]. The lattice parameters were fixed to the experimental values. In confinement an additional vacuum of 17.45 Angstrom was added to the slab in c direction.

[S1] X. Gonze, J.-M. Beuken, R. Caracas, F. Detraux, M. Fuchs, G.-M. Rignanese, L. Sindic, M. Verstraete, G. Zerah, F. Jollet, M. Torrent, A. Roy, M. Mikami, Ph. Ghosez, J.-Y. Raty, D.C. Allan, Computational Materials Science 25 (2002) 478.

[S2] X. Gonze, B. Amadon, P.M. Anglade, J.-M. Beuken, F. Bottin, P. Boulanger, F. Bruneval, D. Caliste, R. Caracas, M. Cote, T. Deutsch, L. Genovese, Ph. Ghosez, M. Giantomassi, S. Goedecker, D. Hamann, P. Hermet, F. Jollet, G. Jomard, S. Leroux, M. Mancini, S. Mazevet, M.J.T. Oliveira, G. Onida, Y. Pouillon, T. Rangel, G.-M. Rignanese, D. Sangalli, R. Shaltaf, M. Torrent, M.J. Verstraete, G. Zérah, J.W. Zwanziger, Computer Physics Communications 180 (2009) 2615.

[S3] J. P. Perdew, K. Burke, M. Ernzerhof, Phys. Rev. Lett. 77 (1996) 3865.

[S4] J. P. Perdew, K. Burke, M. Ernzerhof, Phys. Rev. Lett. 78 (1997) 1396(E).

[S5] C. Hartwigsen, S. Goedecker, J. Hutter, Phys. Rev. B 58 (1998) 3641.
